# 

**Published:** 1847-04-01

**Authors:** 


### CONTENTS

OF THE

### MEDICO-CHIRURGICAL REVIEW,

### APRIL 1, 1847.

I.

I. Histoire de la Medecine depuis son Origine jusqu 'au XIX. Siecle. Par le Dr. P. V. Renouard .. .. .. .. .. .. .. .. .. .. .. .. } 285

II. Essai sur l'Histoire et la Philosophie de la Chirurgie. Par M. Malgaigne }

1. The Age of Foundation .. .. .. .. .. .. .. .. .. .. .. .. 289

A. The Primitive Period .. .. .. .. .. .. .. .. .. .. .. 289

B. The Mystic or Sacred Period .. .. .. .. .. .. .. .. .. 289

C. The Philosophic Period .. .. .. .. .. .. .. .. .. .. 291

D. The Anatomical Period .. .. .. .. .. .. .. .. .. .. 298

2. The Age of Transition .. .. .. .. .. .. .. .. .. .. 310

E. The Greek Period .. .. .. .. .. .. .. .. .. .. .. 310

F. The Arabic Period .. .. .. .. .. .. .. .. .. .. .. 314

II.

Medico-Chirurgical Transactions—concluded from p. 233 .. .. .. .. .. 317
7. An Account of a Case in which the Corpus Callosum, Fornix, and Septum Lucidum were imperfectly formed. By James Paget, F.R.C.S. 317
8. Case of Aneurism presenting some Peculiarities, with Remarks. By Prescott Hewett, Esq. .. .. .. .. .. .. .. .. .. .. .. .. 318
9. Case of Cyanosis of Forty Years' standing, depending upon Congenital Obstruction in the Pulmonary Artery and Patulous Foramen Ovale. By Robert J. Spitta, M.B.. .... .. .. .. .. .. .. .. .. 319
10. On the Ganglionic Character of the Arachnoid Membrane of the Brain and Spinal Marrow. By George Rainey, M.R.C.S. .. .. .. 320
11. An Account of a Case of partial double Monstrosity. By William Acton, Esq. .. .. .. .. .. .. .. .. .. .. .. .. .. 321
12. Some Remarks on Wounded Arteries, Secondary Hæmorrhage and False Aneurisms. By Robert Liston, F.R.S. .. .. .. .. .. .. 322
13. A Case in which a large Tumour was developed in the Substance of the Fifth Nerve and its Ganglion. By James Dixon, Esq. .. .. 327
14. On the Capacity of the Lungs, and on the Respiratory Functions, &c. By John Hutchinson, Surgeon .. .. .. .. .. .. .. .. .. 328
15. Hydatid Cyst, either originating in, or pressing upon the Prostate Gland. By George Lowdell, Surgeon .. .. .. .. .. .. .. 333
16. Cases of Varicocele treated by Pressure, with Observations. By T. B. Curling, Esq. .. .. .. .. .. .. .. .. .. .. .. 333
17. On a particular Derangement of the Structure of the Spleen. By J. B. S. Jackson, M.D., Boston, U.S.A. .. .. .. .. .. .. .. 336
18. On a Luminous Appearance of the Human Eye, and its Application to the Detection of Disease of the Retina and Posterior part of the Eye. By Wm. Cumming, Esq. .. .. .. .. .. .. .. .. .. .. 336
19. Account of a Case in which an Abscess in the Neck communicated by an Ulcerated Opening with the Arch of the Aorta, &c. By George Busk, Esq. .. .. .. .. .. .. .. .. .. .. .. .. .. 339
20. On the Relation between the Constituents of the Food and the Systems of Animals. By R. D. Thomson, M.D. .. .. .. .. .. .. .. 339

III.

I. Lectures on Subjects connected with Clinical Medicine, comprising Diseases of the Heart. By P. M. LATHAM, M.D. &c. .. .. .. .. .. .. .. } 341

II. Traité de Nosographie Medicale. Par J. BOUILLAUD .. .. .. .. .. :

1. Auscultatory and other Phenomena of Chlorosis, &c... .. .. .. .. 343

2. Murmurs in the Veins .. .. .. .. .. .. .. .. .. .. .. .. .. 345

3. Causes and Treatment of Hypertrophy.. .. .. .. .. .. .. .. 347

4. Connection between Hypertrophy and Valvular Lesions .. .. .. 349

5. Atrophy and Softening of the Muscular Substance of the Heart .. 351

6. Consecutive Lesions of the Lungs .. .. .. .. .. .. .. .. .. .. 352

7. Consecutive Lesions of the Liver, Kidneys, &c. .. .. .. .. .. .. 354

8. Cases of Angina Pectoris.. .. .. .. .. .. .. .. .. .. .. .. .. 356

9. Heberden on Angina Pectoris.. .. .. .. .. .. .. .. .. .. .. .. 359

10. Causes of Endocardial Disease .. .. .. .. .. .. .. .. .. .. .. 361

11. Diagnosis between Endocarditis and Pericarditis .. .. .. .. .. 363

12. Various Organic Lesions.. .. .. .. .. .. .. .. .. .. .. .. .. 365

13. Arteritis, its Symptoms, Causes, &c. .. .. .. .. .. .. .. .. .. 366

14. Inflammation of the Aorta and Pulmonary Artery .. .. .. .. .. 368

IV.

I. The Vegetable Kingdom; or the Structure, Classification, and Uses of Plants, illustrated upon the Natural System. By JOHN LINDLEY, Ph. D., F.R.S., F.L.S., &c... .. .. .. .. .. .. .. .. .. .. .. .. .. .. .. } 370

II. Outlines of Structural and Physiological Botany. By ARTHUR HENFREY, F.L.S., &c... .. .. .. .. .. .. .. .. .. .. .. .. .. .. ..

1. Past and Present Mode of Study .. .. .. .. .. .. .. .. .. .. 371

2. Cell-development—Structure.. .. .. .. .. .. .. .. .. .. .. .. 372

3. Morphology—Function .. .. .. .. .. .. .. .. .. .. .. .. .. 374

4. Botanical Geography .. .. .. .. .. .. .. .. .. .. .. .. .. 376

5. Natural and Artificial Systems .. .. .. .. .. .. .. .. .. .. 378

6. Vegetable Chemistry—Terminology .. .. .. .. .. .. .. .. .. 380

7. Properties of the Leguminosæ—Porous Cells? .. .. .. .. .. .. 382

V.

Clinical Facts and Reflections; also, Remarks on the Impunity of Murder in some Cases of presumed Insanity. By THOMAS MAYO, M.D., F.R.S., &c. .. .. 385
1. Petechial Fever, Gastrodynia, Ileus, &c. .. .. .. .. .. .. .. .. 386
2. Periodicity of certain Diseases .. .. .. .. .. .. .. .. .. .. .. 389
3. Treatment of Scarlatina Maligna .. .. .. .. .. .. .. .. .. .. 391
4. Claims of Mesmerism .. .. .. .. .. .. .. .. .. .. .. .. .. 393
5. Recent Medical Heresies . .. .. .. .. .. .. .. .. .. .. .. .. 395
6. How to improve the Art of Medicine .. .. .. .. .. .. .. .. .. 397

VI.

I. Histoire de la Medecine depuis son Origine jusqu'au XIX. Siecle. Par le Dr. P. V. RENOUARD .. .. .. .. .. .. .. .. .. .. .. .. .. .. .. .. } 399

II. Essai sur l'Histoire et la Philosophie de la Chirurgie. Par M. MALGAIGNE, (Continued from page 316) .. .. .. .. .. .. .. .. .. .. .. .. .. ..

3. The Age of Renovation .. .. .. .. .. .. .. .. .. .. .. .. .. 402

G. The Erudite Period—Modern Anatomy .. .. .. .. .. .. .. .. 403

Fernel—Ambrose Paré.. .. .. .. .. .. .. .. .. .. .. .. .. .. 405

New Diseases—The Occult Sciences .. .. .. .. .. .. .. .. .. 407

Cornelius Agrippa—Jerome Cardan—Paracelsus .. .. .. .. .. 408

H. The Reformatory Period .. .. .. .. .. .. .. .. .. .. .. .. .. 410

Progress of Anatomy and Physiology.. .. .. .. .. .. .. .. .. 411

The Vital Forces—Haller .. .. .. .. .. .. .. .. .. .. .. .. .. 414

Progress of Pathology—Morgagni .. .. .. .. .. .. .. .. .. .. 417

Nosological Systems—Therapeutics .. .. .. .. .. .. .. .. .. 418

Progress of Surgery—John Hunter .. .. .. .. .. .. .. .. .. .. 420

Clinical Medicine—Boerhaave and Sydenham .. .. .. .. .. .. 422

Iatro-Chemistry and Iatro-Mechanics .. .. .. .. .. .. .. .. 425

Animism and Vitalism—Stahl .. .. .. .. .. .. .. .. .. .. .. 427

VII.

The Pathological Anatomy of the Human Body, By JULIUS VOGEL, M.D. .. .. 429

1. Proper Mode of Investigation.. .. .. 431

2. Fibrinous Dropsy .. .. 433

3. Pathological Epigeneses .. .. 435

4. Development of Cytoblastema .. .. 437

5. Value of Schwann's Theory .. .. 439

6. Classification of Tumours .. .. 440

7. Malignant Epigeneses .. .. 443

8. Characters of Typhous Deposits .. .. 445

9. Mode of Tubercular Deposit .. .. 446

10. Development of Cancer .. .. 448

11. On Malformations .. .. 451

12. Hermaphroditical Formations .. .. 454

VIII.

I. Dictionary of Practical Medicine. By JAMES COPLAND, M.D. F.R.S.—Parts X. and XI.—Article, Pestilence .. ..

II. A Treatise on the Plague, &c. With Hints on Quarantine. By A. WHITE, M.D. &c. .. .. } 456

1. Distinction between Infection and Contagion .. .. 457

2. Doctrine of Contingent Infection .. .. 459

3. Difference (?) between Spasmodic aud Pestilential Cholera.. .. . 460

4. Epidemic Spasmodic Cholera .. .. 465

5. Rise and Progress of Pestilential Cholera .. .. 467

6. Origin of it in several places .. .. 469

7. Is Pestilential Cholera propagated by Infection? .. .. 470

8. Evidence in the Bombay and Calcutta Reports .. .. 471

9. Evidence in the Madras Report .. .. 467

10. Testimonies to its Non-infectiousness .. .. 478

11. Its Appearance at Astracan and Orenburg .. .. 481

12. Its Appearance at Moscow and St. Petersburg .. .. 482

13. How did it reach this country? .. .. 485

14. Recommendations of our Boards of Health .. .. 487

15. How did it reach the Mauritius? .. .. 488

16. How did it reach North America?.. .. .. 491

17. Cause of its Outbreaks inscrutable.. .. .. 493

18. Extreme Opinions of Dr. Copland.. .. .. 494

19. Essential Infectiousness of the Plague questioned.. .. .. 497

IX.

On Tumours of the Uterus and its Appendages. By THOMAS STAFFORD LEE, M.R.C.S.E. &c. .. .. 498

1. On Fibrous Tumours of the Uterus .. .. 499

2. On the Treatment of Uterine Tumours.. .. .. 501

3. On Encysted Dropsy of the Ovary.. .. .. 503

4. On the Treatment of Ovarian Dropsy .. .. 505

5. On the Diagnosis of Adhesions in Ovarian Dropsy .. .. 506

X.

I. Leçons sur les Phenomenes Physiques des Corps Vivants. By Signor CARLO MATTEUCCI. .. ..
II. Electro-physiological Researches. First Memoir. Muscular Current. By the same .. ..
III. Electro-Physiological Researches. Second Memoir. On the proper Current of the Frog. By the same. .. ..
IV. Electro-Physiological Researches. Third Memoir. On Induced Contractions. By the same. .. .. } 509
1. Porrett's Discovery of Endosmose .. .. 510
2. Agency of Endosmose in Cell-life .. .. 513
3. The Muscular Electric Current .. .. 515
4. On Induced Contractions in Muscles .. .. 517

XI.

I. A Manual of the Principles and Practice of Ophthalmic Medicine and Surgery. By T. Wharton Jones, F.R.S. ..
II. On Cataract, Artificial Pupil, and Strabismus. By F. A. Brett, M.D. F.R.C.S. ..
III. Annales d'Oculistique. Tom. XVI.—XVII. ..
IV. Report of the Progress of Ophthalmic Surgery for 1846. By W. R. Wilde, M.R.I.A. ..
V. Papers on Inflammations of the Eye. By A. Jacob, M.D. F.R.C.S.I. .. } 518
1. Scrofulous and Rheumatic Ophthalmia .. 520
2. Gonorrhœal Ophthalmia .. 523
3. Congenital Tumours of the Conjunctiva .. 526
4. Cataract, relative value of different Operations for .. 527
5. The Operation for Strabismus .. 529

XII.

I. A Treatise on the Inhalation of the Vapour of Ether for the Prevention of Pain in Surgical Operations. By James Robinson, Surgeon-Dentist ..
II. Gazette Medicale .. } 530

XIII.

Practical Observations on some Diseases of the Stomach and Alimentary Canal. By James Alderson, M.D., F.R.S. &c. &c. .. 537
Different Forms of Carcinomatous Disease .. 539

XIV.

Experimental Researches on the Post-mortem Contractility of the Muscles, &c. By Bennet Dowler, M. D. .. 544

XV.

The Microscopic Anatomy of the Human Body in Health and Disease. By Arthur Hill Hassall .. 547

XVI.

Body and Soul; or Life, Mind, and Matter considered as to their peculiar Nature and combined Condition in Living Things, &c. By George Redford, M.R. C.S. &c. .. 548

XVII.

On the Relations of the Physician to the Sick, to the Public, and to his Colleagues. By the late C. W. Hufeland, M.D .. 548

XVIII.

An Inquiry into the Action of Mercury on the Living Body. By Joseph Swan .. 549

XIX.

An Essay on the Tongue in Functional Derangement of the Stomach and Bowels, and on the appropriate Treatment, &c. By Edward Williams, M.D. &c. .. 550

XX.

On Indigestion, and certain Bilious Disorders conjoined with it, &c. By G. C. Child, M.D. &c. .. 550

XXI.

Lectures and Observations on Clinical Surgery. By Andrew Ellis .. 552

XXII.

Practical Observations on Mineral Waters and Baths, &c. By Edwin Lee, Esq. .. 555

XXIII.

On the Correlation of Physical Forces. By W. R. Grove, M.A., F.R.S.. &c. .. 557

XXIV.

Researches into the Physical History of Mankind. By James Cowle Prichard, M.D. &c. .. 559

XXV.

On the Mechanism of Respiration. By Francis Sibson, Esq. .. 560

XXVI.

The London and Provincial Medical Directory, 1847 .. 560

